# Circumstantial risk factors for death after intensive care unit-to-unit inter-hospital transfer—a Swedish registry study

**DOI:** 10.1186/s13049-025-01325-2

**Published:** 2025-01-29

**Authors:** Jesper Sternley, Karl Stattin, Max Petzold, Jonatan Oras, Christian Rylander

**Affiliations:** 1 Anaesthesiology and Intensive Care, Department of Surgical Sciences, Uppsala University, 715 85 Uppsala, Sweden; 2 Department of Anaesthesiology and Intensive Care Medicine, Clinical Sciences, University of Gothenburg and Sahlgrenska University Hospital, Gothenburg, Sweden; 3 School of Public Health and Community Medicine, Institute of Medicine, University of Gothenburg, Gothenburg, Sweden

**Keywords:** Intensive care unit, Intensive care transport, Patient transfer, Mortality

## Abstract

**Background:**

Unit-to-unit transfer of critically ill patients infers hazards that may cause adverse events. Circumstantial factors associated with mortality after intensive care include days in the ICU, night-time or weekend discharge and capacity transfer as compared to other reasons for transfer. Distance travelled may also constitute an indirect risk. The aim of this study was to assess potential associations between these circumstantial factors and the risk of death 30 days after transfer.

**Methods:**

Data from 2015 to 2019 was retrieved from the Swedish Intensive Care Registry. Logistic regression was used for risk analysis.

**Results:**

Among 4,327 patients, 965 (22%) were deceased 30 days after transfer. 1351 patients undergoing capacity transfer had a higher morbidity than patients transferred for other reasons. Using univariable logistic regression, days spent in the referring ICU before transfer, capacity transfer as compared to clinical transfer and repatriation as well as SAPS3 in the receiving ICU were associated with a higher risk of death at 30 days. However, after multivariable regression with adjustment for ICD-10 diagnosis and Standardised Mortality Rate in the receiving ICU, these associations were lost.

**Conclusion:**

Our results suggest that inter-hospital transfer is safe to carry out at any time of day and over shorter as well as longer distances.

**Supplementary Information:**

The online version contains supplementary material available at 10.1186/s13049-025-01325-2.

## Background

Transfer of a critically ill patient during uninterrupted intensive care introduces numerous practical hazards [1, 2]. Adverse events, such as serious hypotension or ventilation difficulties may occur and hemodynamic instability before transfer is an important risk factor for complications *en route* [3]. Notwithstanding, with specialised staffing most patient- or equipment-associated incidents can be detected early and handled safely without impact on mortality [4, 5].

However, circumstantial factors not directly related to patient care may be more difficult to attenuate. The number of days spent in intensive care before transfer is a marker of disease severity associated with risk of death after transfer between intensive care units (ICUs) [6, 7]. Night-time ICU discharge has been associated with increased risk of readmission and hospital death in several studies [8]. A low weekend staff-to-patient ratio may be associated with increased mortality [9]. Intuitively, longer distance with extended time in transit during inter-hospital transfer may infer more problems than a shorter transfer.

Notwithstanding, unit-to-unit referral of critically ill patients is part of many health care systems, necessitated for different reasons. In Sweden, transfers between ICUs are registered and categorised in the Swedish Intensive Care Registry. A recent study, using data from all transfers registered over three years, found that patients submitted to transfer due to lack of resources (capacity transfer), suffered a higher risk of death at 30, 90 and 180 days compared to patients transferred for other reasons [10].

The primary aim of this study was to analyse, for all inter-hospital transfers, whether any of the circumstantial factors ICU length of stay before transfer, night-time or weekend transfer, distance travelled or the capacity transfer category was associated with the risk of death 30 days after transfer. Secondly, we wanted to analyse for inter-hospital capacity transfer if any of the other circumstantial factors was associated with an increased risk of death at 30 days.

## Methods

This was a cohort study based on data from the Swedish Intensive Care Registry; www.icuregswe.org. After ethical approval (Swedish Ethical Review Authority, Dnr 2020–07089), data retrieval was formally applied for and granted by the board of the registry. In the data set, individual patients were pseudomised, only identifiable by a code number for which the key was not released from the registry. The manuscript was prepared in line with the guidelines for Strengthening the Reporting of Observational Studies in Epidemiology (STROBE) [11].

### Setting

Swedish ICUs are categorised according to their capacity to deliver complex care. Category I ICUs in local hospitals offer basic intensive care including mechanical ventilation. Category II ICUs are found in regional hospitals providing relatively complex treatments like renal replacement therapy while category III ICUs are located in university hospitals housing complex surgery and medical subspecialties. In fact, Swedish ICUs in all local hospitals are categorised as Category I, those in county hospitals as Category II, and university hospital ICUs as Category III. Inter-hospital transfer of intensive care patients is mainly carried out by ambulance services using vehicles furnished with modern technical equipment [12]. Planned, secondary transfers can be organised with a special vehicle designed for delivering intensive care during transfer. The most urgent transfers to a higher level of care may need to use any ambulance available even if the technical equipment is more basic. For distances exceeding 250 kms, aviation services are routinely used. The necessary pre-hospital competency in intensive care or anaesthesia is provided either by a proficient prehospital team or by a nurse or a physician from the referring hospital. Some road ambulance organisations and all air services use their own, specially trained physicians. There are no standards issued by Swedish authorities, but the Swedish Society of Anaesthesia and Intensive Care (SFAI); www.sfai.se, published professional recommendations on intensive care transfers in 2015 to which Swedish hospitals offering intensive care service are expected to adhere.

### Patients

Adults aged ≥ 18 years treated between January 1st 2015 and December 31st 2019 were included in the data retrieval from the Swedish Intensive Care Registry. Patients with a registered first transfer between ICUs were eligible for inclusion. Subsequent or incompletely registered transfers, patients transferred within the same hospital or to neurosurgical or thoracic ICUs and patients lacking SAPS3 (Simplified Acute Physiology Score 3) data or survival status were excluded. However, diagnoses usually treated in such units were accepted if they were registered during intensive care in a general ICU from which the patient had been transferred. Requisites for a patient unit-to-unit transfer to be confirmed included the involved ICUs to have mutual documentation of each other as referring and receiving units and to have entered matching data for patient identity, transfer date, time and category into the registry. Follow-up ended on June 22nd, 2021, 18 months after the last transfer.

### Data

The Swedish Intensive Care Registry is an open quality registry run as an association with 83 member-ICUs reporting in average 45,896 yearly admittances 2015–2019. The national quality indicators compulsory for ICUs to report include unit-to-unit patient transfer categorised as (1) “clinical transfer” when due to need for specialised care not available in the referring hospital, (2) “capacity transfer” when due to a lack of resources in the referring ICU or (3) “repatriation” when patients are returned or forwarded to the ICU closest to their home address. The variables included in the data retrieval were: age, sex, date and time of admission, SAPS3, surgical status (elective surgery, acute surgery, no surgery), primary ICU ICD-10 diagnosis (ICD-10; International Statistical Classification of Diseases and Related Health Problems), date and time of discharge, discharge destination (specified ward or ICU), transfer category and the time of death for deceased patients which is automatically imported from the Swedish Population Register (Supplemental table S1). The ICD-10 diagnoses were categorised depending on disease and organ system involved as described previously and tested for association with the risk of death at 30 days (Supplemental table S5) [13]. Standardised Mortality Rate (SMR), a measure of ICU performance defined by the actual mortality divided by the expected mortality, according to the SAPS3 in the receiving ICU, was collected from the Swedish Intensive Care Registry website; www.icuregswe.org.

### Exposure

The following potential risk factors for patients submitted to inter-hospital transfer were chosen for predictors: (1) The duration of intensive care before transfer, defined as number of commenced full days (24 h) calculated from the difference in hours between admission and discharge in the referring hospital, (2) Night-time transfer, defined as discharge from referring hospital between 10:00 PM and 7:00 AM [14], (3) Weekend transfer, defined by Saturdays and Sundays. Capacity transfer, defined by lack of resources in the referring ICU [10] and (4) the direct distance travelled, expressed in kilometres (km), between the referring and the receiving hospital and calculated with a trigonometric formula using coordinates retrieved from Google Maps (Table S6) [15]:

$$Distance = ACOS\left( {COS\left( {RADIANS\left( {90 - Lat1} \right)} \right) \, * \, COS\left( {RADIANS\left( {90 - Lat2} \right)} \right) \, + \, SIN\left( {RADIANS\left( {90 - Lat1} \right)} \right) \, * \, SIN\left( {RADIANS\left( {90 - Lat2} \right)} \right) \, * \, COS\left( {RADIANS\left( {Long1 - Long2} \right)} \right)} \right) \, * \, 6371.$$

The distance was also defined as long or short by dichotomization at 25 kms, chosen to represent a maximum distance between ICUs within major cities with several hospitals, thus constituting a separation between urban and rural trajectories.

SAPS3 at arrival in the receiving ICU was added to the predictors as it is designed to be associated with the risk of death after intensive care [16].

### Outcome

The outcome of interest was survival status 30 days after transfer and its potential association with selected risk factors referenced above. The time point was chosen over long-term survival because of its data robustness and its vicinity in time to the transfer episode.

### Statistical analysis

Symmetrically distributed continuous variables are presented as mean (standard deviation; SD) with the two-sided Student’s t-test used for comparison between groups. Non-symmetrically distributed continuous variables are presented as median (first and third quartile; Q1;Q3) with the Mann–Whitney U-test used for comparison between two groups and the Kruskal–Wallis analysis of ranks applied to three groups. The respective Chi-square or Fisher´s exact test was used for comparison of binary variables.

The association between the outcome and the selected risk factors was assessed both for the entire cohort of patients submitted to inter-hospital transfer and for the patients having undergone capacity transfer. The risk of death, expressed as the event odds ratio (OR) with corresponding 95% confidence interval (CI) associated with a certain exposure, was assessed by applying univariable and multivariable logistic regression, respectively. Each predictor was first tested alone, and predictors with a p-value < 0.1 were subsequently included in a multivariable analysis with premeditated adjustment for ICD-10 diagnosis and SMR in the receiving ICU, which can be expected to be associated with patient outcome. In the multivariable analysis, p < 0.05 was considered statistically significant. The statistical package SPSS (v24.0, IBM Statistics, IBM Corp, Armonk, NY, USA) was used for all analyses.

## Results

### Transfers

**Fig. 1:**
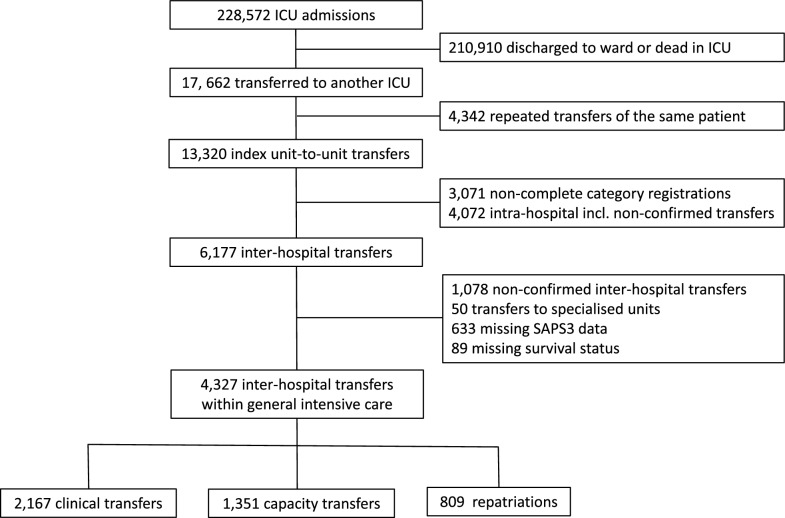
Study flow chart

During the 5-year study period, there were 228,572 ICU admissions recorded in the Swedish Intensive Care Registry and 17,662 unit-to-unit transfers occurred. Among the 4327 patients with correctly registered inter-hospital transfers, 2167 had been submitted to clinical transfer, 1351 to capacity transfer and 809 to repatriation (Fig. 1). The age was 65 (52;73) years and the proportion of women was 37%. The number of days spent in the referring ICU before transfer was 1 (0;3) and the proportion of night-time transfer was 16%. The distance travelled was 58.3 (18.8:108) kilometres with 71% of the transfers being longer than 25 kms. The SAPS3 was 60 (50;70) in the referring ICU and 60 (51:70) in the receiving ICU and both scores were higher in the patients submitted to capacity transfer than in the patients having undergone clinical transfer or repatriation (Kruskal–Wallis rank test; p < 0.001). Data partitioned on categories of inter-hospital transfers appear in Table 1.

**Table 1:** Patient and unit-to-unit inter-hospital transfer characteristics

		Transfer category		
Domain	Variable	Clinical n = 2167	Capacity n = 1351	Repatriation n = 809
Demographics	Age years, median (Q1;Q3)	62 (48;72)	68 (57;76)	66 (53;74)
	Women, n (%)	821 (38)	545 (40)	256 (31)
Transfer volume	2015, n (%)	401 (18)	222 (16)	170 (21)
	2016. n (%)	438 (20)	282 (21)	146 (18)
	2017, n (%)	407 (19)	292 (22)	146 (18)
	2018, n (%)	436 (20)	280 (21)	191 (20)
	2019, n (%)	485 (22)	275 (20)	186 (23)
Referring ICU	Acute admission, n (%)	2125 (98)	1319 (98)	754 (93)
	SAPS3 total, median (Q1;Q3)	57 (47;67)	64 (56;74)	61 (52;71)
	SAPS3% EMR, median (Q1;Q3)	15 (5;32)	26 (14;47)	21 (9;40)
	SMR, median (Q1;Q3)	1.00 (0.88;1.10)	0.94 (0.88;1.00)	0.91 (0.84;1.00)
	No surgery, n (%)	1919 (89)	1160 (86)	469 (58)
	Acute surgery, n (%)	204 (9)	151 (11)	240 (30)
	Elective surgery, n (%)	44 (2)	40 (3)	100 (12)
	Hours in the ICU before transfer, median (Q1;Q3)	9 (3;33)	50 (17;131)	75 (39;178)
	Days in the ICU before transfer, median (Q1;Q3)	0 (0;1)	2 (0;5)	3 (1;7)
	Day time discharge, n (%)	1692 (78)	1142 (85)	784 (97)
	Night-time discharge, n (%)	475 (22)	209 (15)	25 (3)
	Weekend transfer, n (%)	577 (27)	363 (27)	219 (27)
Transfer distance	Direct distance in km, median (Q1;Q3)	70,0 (19,9;117)	36,3 (6,83;58,4	84,8 (59,5;139)
	Short distance < 25 km, n (%)	570 (26)	636 (47)	56 (7)
	Long distance > 25 km, n (%)	1597 (74)	715 (53)	753 (93)
Receiving ICU	SAPS3 total, median (Q1;Q3)	59 (50;69)	62 (54;72)	58 (49;69)
	SAPS3% EMR, median (Q1;Q3)	18 (7;36)	22 (11;42)	17 (6;36)
	SMR, median (Q1;Q3)	0.91 (0.80;0.95)	0.94 (0.88;1.07)	1.00 (0.91;1.08)
Main ICU diagnosis	**n/missing**	**2121/46**	**1346/5**	**806/3**
	Infection/sepsis, except pneumonia, n (%)	359 (17)	205 (15)	51 (6)
	Malignancy, n (%)	21 (1)	4 (0)	13 (2)
	Hematology, n (%)	16 (1)	4 (0)	2 (0)
	Endocrinal disease, n (%)	6 (0)	32 (2)	6 (1)
	Intoxication, n (%)	19 (1)	67 (5)	5 (1)
	Neurological disorder, n (%)	67 (3)	74 (5)	32 (4)
	Cardiac disease, n (%)	80 (4)	40 (3)	31 (4)
	Cardiac arrest, n (%)	58 (3)	156 (12)	78 (10)
	Subarachnoid haemorrhage, n (%)	113 (5)	1 (0)	30 (4)
	Cerebrovascular event, n (%)	196 (9)	23 (2)	68 (8)
	Aortic rupture/dissection, n (%)	41 (2)	4 (0)	26 (3)
	Peripheral aortic disease, n (%)	14 (1)	3 (0)	7 (1)
	Musculoskeletal disorder, n (%)	0 (0)	0 (0)	2 (0)
	Shock, undefined, n (%)	20 (1)	12 (1)	3 (0)
	Respiratory infection, including pneumonia, n (%)	80 (4)	263 (20)	62 (8)
	Airway disorder, n (%)	27 (1)	14 (1)	5 (1)
	COPD/asthma/other respiratory disease, n (%)	99 (5)	266 (20)	83 (10)
	Acute renal failure/urological disease, n (%)	37 (2)	22 (2)	22 (3)
	Acute abdomen, n (%)	174 (8)	36 (3)	20 (2)
	Liver failure, n (%)	52 (2)	11 (1)	10 (1)
	Pancreatitis/cholecystitis, n (%)	32 (2)	25 (2)	9 (1)
	Psychiatric disease, n (%)	1 (0)	0 (0)	1 (0)
	Major haemorrhage, n (%)	11 (1)	1 (0)	3 (0)
	Trauma, n (%)	331 (16)	40 (3)	97 (12)
	Surgical complications, n (%)	54 (3)	12 (1)	26 (3)
	Transplantation, n (%)	2 (0)	0 (0)	1 (0)
	Isolated traumatic brain injury, n (%)	193 (9)	22 (2)	90 (11)
	Pregnancy related disorders, n (%)	1 (0)	0 (0)	0 (0)
	Postoperative care, n (%)	17 (1)	9 (1)	23 (3)

ICU; Intensive Care Unit, SAPS3; Simplified Acute Physiology Score 3, EMR; Estimated Mortality Rate, SMR; Standardised Mortality Rate, km; kilometre

**Fig. 2:**
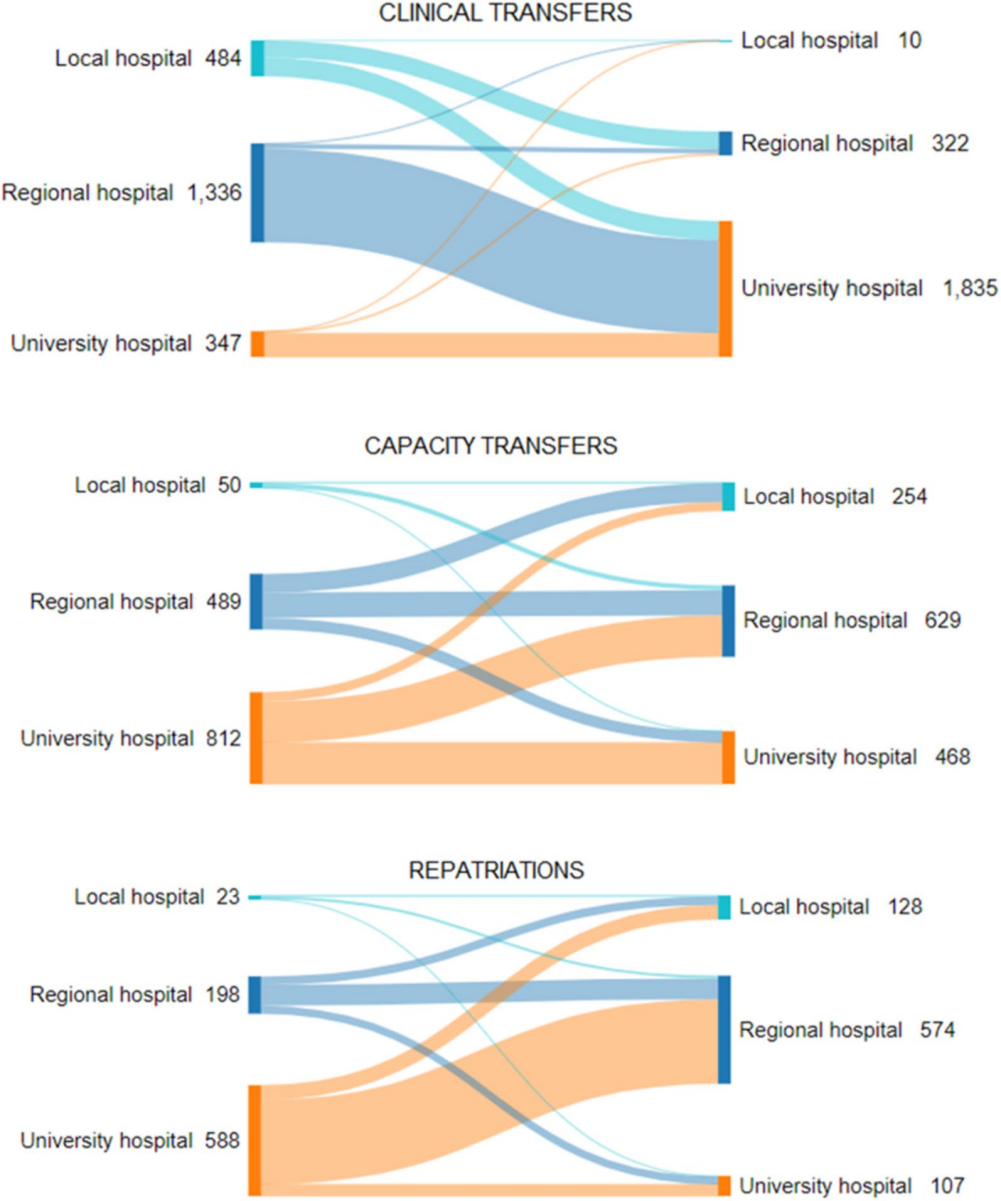
Flow of inter-hospital transfers: referring hospital to the left, receiving hospital to the right

**Fig. 3:**
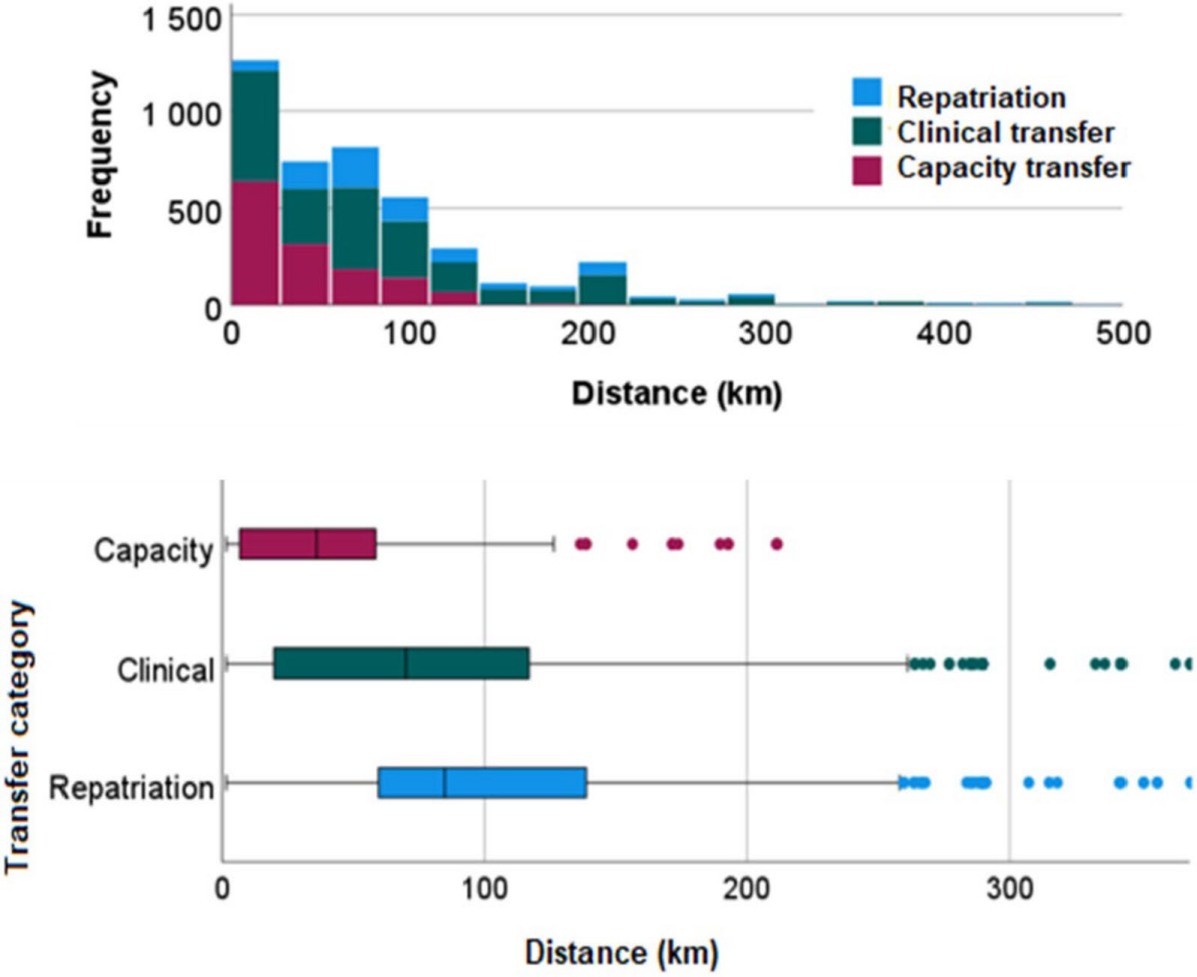
Number of transfers by distance; Top: frequency histogram. Bottom: Distances by box-plots with mean, inter-quartile and full range and outliers defined as > 1.5 × first or third quartile

The majority of referring ICUs were located in regional hospitals and the receiving ICUs in university hospitals. Few patients were transferred between ICUs in community hospitals. Most clinical transfers were destined to university hospitals while most repatriations departed from them. The capacity transfers occurred between all ICU categories but very few out of local hospitals (Fig. 2). The number of transfers by distance and the distance travelled for different categories appear in Fig. 3.

### Risk of death

At 30 days, the proportion of deceased patients was 24% after repatriation, 21% after clinical transfer and 28% after capacity transfer. These proportions had increased by 90 and 180 days but with preserved relations between the transfer categories (Table 2).

**Table 2:** Death rates after unit-to-unit inter-hospital transfer

	Transfer category		
Status	Clinical n = 2167	Capacity n = 1351	Repatriation n = 809
Deceased in receiving ICU	192 (9)	132 (10)	72 (9)
Deceased at 30 days, n (%)	450 (21)	381 (28)	134 (24)
Deceased at 60 days, n (%)	532 (25)	431 (32)	223 (28)
Deceased at 90 days, n (%)	558 (26)	458 (34)	236 (29)
Deceased at 180 days, n (%)	615 (28)	491 (36)	250 (31)

ICU; Intensive Care Unit

Using univariable logistic regression, days spent in the referring ICU before transfer, capacity transfer as compared to clinical transfer and repatriation as well as SAPS3 in the receiving ICU were all associated with a higher risk of death at 30 days (Table 3a). In the multivariable logistic regression with adjustment for ICD-10 diagnosis and SMR in the receiving ICU, there was no remaining statistical association between ICU days before transfer or capacity transfer and the risk of death 30 days later (Table 3b). Similar results were seen for the selected risk factors and death at 90 or 180 days (Tables S2 and S3) with the exception that distance (km travelled) turned out significant in the univariable analysis but did not remain so in the multivariable analysis for 180 days.

**Table 3:** Association between selected predictors and the risk of death 30 days after unit-to-unit inter-hospital transfer

3a. Univariable logistic regression (n = 4,327)			
Predictor	OR	95% CI	p-value
Days in the ICU before transfer	1.01	1.00–1.02	0.047*
Night-time transfer (reference: day-time)	0.94	0.77–1.13	0.502
Weekend transfer (reference: weekday)	0.94	0.80–1.10	0.411
Distance, per kilometer (km)	0.99	0.99–1.00	0.178
Long transfer, > 25 km (reference: short < 25 km)	0.90	0.77–1.04	0.156
Capacity transfer (reference: clinical)	1.50	1.28–1.75	< 0.001*
Capacity transfer (reference: repatriation)	1.25	1.02–1.52	0.032*
SAPS3 receiving ICU, per point	1.08	1.07–1.08	< 0.001*

3b. Multivariable logistic regression adjusted for ICD-10 diagnosis and SMR in the receiving ICU (n = 4,242; 85 missing)			
Variable	OR	95% CI	p-value
Days in the ICU before transfer	1.00	0.99–1.02	0.666
Capacity transfer (reference: clinical)	1.15	0.93–1.42	0.189
Capacity transfer (reference: repatriation)	1.12	0.88–1.43	0.359
SAPS3 receiving ICU, per point	1.08	1.07–1.09	< 0.001*

3a: ICU; Intensive Care Unit, SAPS3; Simplified Acute Physiology Score 3, *denotes a significant p-value (<0.1)

3b: SMR; Standardised Mortality Rate, ICU; Intensive Care Unit, SAPS3; Simplified Acute Physiology Score 3, *denotes a significant p-value (< 0.05)

In the univariable logistic regression analysis of patients submitted to capacity transfers, only SAPS3 was associated with the risk of death at 30 days (Table 4). Similar results were obtained for 90 and 180 days (Tables S4a and S4b).

**Table 4:** Association between selected predictors and the risk of death 30 days after unit-to-unit inter-hospital capacity transfer

Univariable logistic regression (n = 1,352)			
Predictor	OR	95% CI	p-value
Days in the ICU before transfer	1.00	0.99–1.02	0.638
Night-time transfer (reference: day-time)	0.95	0.68–1.32	0.746
Weekend transfer (reference: weekday)	0.97	0.75–1.27	0.852
Distance, per kilometer (km)	0.99	0.99–1.00	0.689
Long transfer, > 25 km (reference: short < 25 km)	0.84	0.66–1.07	0.159
SAPS3 receiving ICU, per point	1.07	1.06–1.08	< .0.001*

ICU; Intensive Care Unit, SAPS3; Simplified Acute Physiology Score 3

*Denotes a significant p-value (< 0.1)

## Discussion

In this registry study of intensive care patients submitted to inter-hospital unit-to-unit transfer during five years in Sweden, we did not find statistically significant associations between ICU length of stay, night-time or weekend transfer, transfer distance or transfer due to lack of resources and the risk of death 30 days later. Previous results are conflicting but comparison between studies in this field is difficult as patient populations differ, transfers may be categorised differently, the standards of care may diverge between the referring and the receiving unit and different time points are chosen for analysis of mortality [17].

Up to ten days of stay within intensive care has been associated with progressively increased long-term mortality after hospital discharge [7, 18]. We reasoned that ICU stay before transfer could be a risk factor for death after transfer, but did not find such an association. The fact that the number of days spent in the ICU before transfer was low may have affected this finding.

Night-time discharge from intensive care has been associated with higher mortality and increased risk of re-admission in different settings [8, 19]. This has been attributed to a premature discharge of the patient to a lower level of care due to strain on ICU capacity [14]. In our cohort, night-time transfer was not associated with a higher risk of death than day-time transfer. A potential explanation may be that night-time unit-to-unit transfer, with intensive care being uninterrupted during transfer and continued in the new ICU, does not constitute the same risk as night-time discharge to an ordinary ward that offers a lower level of care. Neither was transfer on weekends, when staffing may be expected to be lower [9], a significant risk factor.

We did not find any statistically significant association between the number of kilometres or distances > 25 kms and risk of death at 30 days. To our knowledge, the present study is the first analysis of a potential association between the exact transfer distance and subsequent risk of death in an adult mixed cohort. It was previously demonstrated in Finland that patients with severe respiratory failure could be transferred over long distances without major complications [20]. From France, it was reported that even the most serious cases of respiratory failure in need of veno-venous extracorporeal membrane oxygenation (V-V ECMO) could be transferred without deleterious complications [21]. In New Zealand, a paediatric study showed that children transferred over long distances to intensive care did not have a higher risk- adjusted mortality compared to non-transferred children [22]. These studies emphasise that their results were valid for transfers executed by specialised teams, which is also representative for safe airborne transfers of critically ill patients [23]. Our results and the literature taken together suggest that modern means of transfer and competent staffing enable uninterrupted intensive care and safe transfer over long distances.

We also found that, with adjustment for ICD-10 diagnosis and SMR in the receiving ICU, the risk of death at 30 days was not higher with capacity transfer as compared to the other categories. These results differ from a recently published Swedish study based on similar registry data, demonstrating a higher adjusted mortality with capacity and clinical transfer as compared to repatriation [10]. There are some methodological differences between the studies. We only included patients exposed to inter-hospital transfer while the other cohort consisted of both intra- and inter-hospital transfers. The larger cohort of 11,176 patients in the other study also included a number of cases that would have been excluded in our study for reasons such as non-confirmed unit-to-unit transfers and incomplete registrations. Furthermore, we adjusted for morbidity using ICD 10 diagnosis and for the statistical risk of death in the receiving ICU using SMR. Although our cohort of 4,327 patients was smaller, the result agrees with two other recent Swedish studies that did not show a higher mortality after capacity transfer as compared to no transfer [13, 24]. Similarly, a British and a Canadian study with matched controls, did not show increased mortality after transfer although the hospital stay was longer after capacity transfer in the British cohort [25, 26]. In a study of short-term mortality, within 24 h after inbound transfer, death was associated with specific diagnoses but not the transfer itself [27]. Our interpretation of these studies taken together is that co-morbidities and the seriousness of the actual state of the patient are the predominant predictors of patient survival, unrelated to the transfer itself.

In summary, it seems that unit-to-unit transfer could be performed without patient risk of death being associated with the number of days spent in the ICU before transfer, the hour of day, the day of the week or the time spent in transfer over different distances. Considering that unit-to-unit transfers employ modern facilities with additional crew members assigned to the care of the patient, it could be argued that the patient is provided uninterrupted intensive care during transfer. It may be that sedation and balancing vasopressors have to be increased consequent to physical movements, vibration and noise, but the practical hazards have been shown to be identified and handled by the crew without affecting patient outcome [4, 5]. This is of clinical importance since the number of available beds for critical care in Sweden is among the lowest in Europe and patients are often transferred between intensive care units due to an acute lack of capacity [28].

### Methodological aspects

For study period, we chose 2015–2019 in order to have a cohort representative of non-pandemic conditions. Among several potential risks during transfer, we selected factors not related to the medical status of the patient but to the process of transfer. The exception to this was the SAPS3, which was included as it is designed to be indicative of the health status of the patient and may thus be used to adjust for disease severity together with ICD-10 diagnosis and SMR which both can be expected to influence outcome [24]. As SAPS3 includes age and major co-morbidities we did not separately adjust for these to avoid over-fitting of the analysis.

This study focused on inter-hospital transfers because intra-hospital transfers between intensive care units or from the ICU to procedures are short and usually involve the same persons caring for the patient in the ICU, while inter-hospital transfers are more complex, exposing the patient to a longer time in transit, different means of transfer and staff handovers. We considered the transfer distance a rough indicator of the time in transit, especially travel for more than 25 kms, which we chose as a proxy for dichotomisation between transfer to a hospital within the same metropolitan area and transfer to a hospital in a different city. As for kilometres travelled, we could not get access to enroute data from the aircraft or vehicles used for transfer and we chose an objective formula that did not yield the exact road distance but the direct surface distance between the actual hospital [15]. This can be expected to be inexact in urban areas with numerous road connections but with increasing distance, the difference between road and direct distances should diminish.

### Limitations

Study limitations include the retrospective design and the use of registry data impossible to verify in medical records. The Swedish Intensive Care Registry covers all Swedish general ICUs and transfers are registered according to a well-defined classification, well recognised within the health care organisation. As for data quality, the variables mandatory to register are well defined and the amount of missing data was negligible, at most 2% of ICU diagnoses (Table 1) but there was no way of verifying the correctness of the transfer category. The persons responsible for entering data into the registry have varying experience and transfer category may be understood differently between ICUs. To increase specificity of the case selection for inclusion, identical reasons for transfer registered both by the referring and the receiving ICUs was a mandatory criterion, but excluded transfers may actually have been executed in spite of incomplete entries in the registry. Furthermore, the dataset included variables registered while the patients were in the ICUs but as incidents during transfer are underreported, non-registered events with a potential effect on morbidity may have occurred [29].

Our analysis is limited to showing that we did not find a statistically significant association between selected risk factors and death after transfer and there may be other, unmeasured confounders strongly associated with mortality. An earlier review that included five studies of primary and secondary transfers conducted by air and ground transfers, concluded that more data was needed to determine whether inter-facility transfer of intensive care patients leads to increased mortality [30]. The choice of mortality as the outcome is also a limitation since it is known that transfer of a critically ill patients is associated with negative experiences by patients, families and health care professionals which we did not have data to analyse [31, 32].

## Conclusion

For unit-to-unit inter-hospital intensive care transfers in Sweden, during five years preceding the Covid-19 pandemic, we did not find a statistically significant association between duration of intensive care before transfer, night-time or week-end transfer, transfer distance or category of transfer and the risk of death 30 days after transfer. This suggests that modern transfer between ICUs is safe to carry out at any time of day and over shorter as well as longer distances. It should however be made clear that we report an analysis on the population level. A few serious adverse outcomes would not affect the results but they would nonetheless be unacceptable to the individual patient, especially if the reason for transfer was the lack of capacity for other patients in the referring ICU.

## Supplementary Information

Supplementary material 1.

## Data Availability

Due Swedish legislation the database is not available to the public. Questions about the data can be addressed to the corresponding author.

## Abbreviations

CI: Confidence Interval
COPD: Chronic Obstructive Pulmonary Disease
Dnr: Diary number
SAPS3: Simplified Acute Physiology Score version 3
IBM: International Business Machines Corporation
ICD-10: International Classification of Diseases Tenth Revision
ICU: Intensive Care Unit
OR: Odds Ratio
Q1;Q3: First Quartile;Third Quartile
SD: Standard Deviation
SFAI: Swedish Society of Anaesthesia and Intensive Care
SMR: Standardised Mortality Rate
SPSS: Statistical Package for the Social Sciences
STROBE: Strengthening the Reporting of Observational Studies in Epidemiology
<: Less than
>: More than
