# Supplementary material for: Circumstantial risk factors for death after intensive care unit-to-unit inter-hospital transfer—a Swedish registry study
Source: Scand J Trauma Resusc Emerg Med. 2025 Jan 29;33:14. doi: 10.1186/s13049-025-01325-2 (PMC11780853; doi:10.1186/s13049-025-01325-2)
Supplement: Supplementary file 1 — Supplementary material 1. [file 13049_2025_1325_MOESM1_ESM.docx]

**Supplement**

*Potential risk factors for death after intensive care unit-to-unit inter-hospital transfer – a Swedish registry study*

**Table S1**

**List of variables**

Care identification number

Patient identification number

Hospital

Unit

Type of care

Age

Gender

Surgery

Acute admission

Admission through

Reason for admission

Time of admission

Time of discharge

Discharged to

Reason for discharge

Intrahospital survival

Time of death

NEMS score

VTS score

Treatment strategy

SAPS3 score

SAPS3 EMR

SAPS3 cancer therapy

SAPS3 chronic heart failure

SAPS3 haematological malignancy

SAPS3 cirrhosis

SAPS3 AIDS

SAPS3 cancer

SAPS3 time in hospital before admission

SAPS3 previous care

SAPS3 type of surgerSAPS3 therapy

SAPS3 acute nosocomial infection

SAPS3 acute deep respiratory infection

SAPS3 GCS

SAPS3 GCS eyes

SAPS3 GCS verbal

SAPS3 GCS motor function

SAPS3 RLS85

SAPS3 body temperature maximum

SAPS3 heart frequency maximum

SAPS3 systolic blood pressure minimum

SAPS3 creatinine maximum

SAPS3 bilirubin maximum

SAPS3 leukocytes maximum

SAPS3 pH minimum

SAPS3 thrombocytes minimum

SAPS3 FiO2

SAPS3 PaO2

SAPS3 ventilator

SAPS3 only observation

SAPS3 cardiovascular

SAPS3 neurological

SAPS3 liver

SAPS3 gastrointestinal

SAPS3 renal

SAPS3 respiratory

SAPS3 hematologic

SAPS3 metabolic

SAPS3 trauma

SAPS3 other

Type of identification number

Follow up 2021-06-23

Deceased

Date for deregistration

Deregistration number of days after admission

Mortality 30 days

Mortality 60 days

Mortality 90 days

Mortality 180 days

**Table S2**

**Association between selected predictors and the risk of death
90 days after unit-to-unit inter-hospital transfer**

| **S2a. Univariable logistic regression (n=4,327)** |  |  |  |
| --- | --- | --- | --- |
| **Predictor** | **OR** | **95% CI** | **p-value** |
| Days in the ICU before transfer | 1.02 | 1.01 – 1.03 | 0,002* |
| Night-time transfer (reference: day-time) | 0.93 | 0.78 – 1.11 | 0.408 |
| Week-end transfer (reference: weekdays) | 0.98 | 0.84 – 1.13 | 0.742 |
| Distance, per kilometer (km) | 0.99 | 0.99 – 1.00 | 0.098* |
| Long transfer, >25 km (reference: short <25 km) | 0.92 | 0.80 – 1.07 | 0.274 |
| Capacity transfer (reference: clinical) | 1.48 | 1.28 – 1.72 | <0.001* |
| Capacity transfer (reference: repatriation) | 1.25 | 1.03 – 1.50 | 0.023* |
| SAPS3 receiving ICU, per point | 1.08 | 1.07 – 1.09 | <0.001* |
| ICU; Intensive Care Unit, SAPS3; Simplified Acute Physiology Score 3  *denotes a significant p-value (<0.1) | | | |
|  |  |  |  |
| **S2b. Multivariable logistic regression adjusted for ICD-10 diagnosis and SMR**  **in the receiving ICU (n=4,242; 85 missing)** | | | |
| **Variable** | **OR** | **95% CI** | **p-value** |
| Days in the ICU before transfer | 1.01 | 0.99 – 1.02 | 0.296 |
| Distance, per kilometer (km) | 1.00 | 0.99 – 1.00 | 0.617 |
| Capacity transfer (reference: clinical) | 1.17 | 0.95 – 1.43 | 0.135 |
| Capacity transfer (reference: repatriation) | 1.14 | 0.90 – 1.44 | 0.291 |
| SAPS3 receiving ICU, per point | 1.08 | 1.07 – 1.09 | <0.001* |
| SMR; Standardised Mortality Rate, ICU; Intensive Care Unit, SAPS3; Simplified Acute Physiology Score 3, *denotes a significant p-value (<0.05) | | | |

**Table S3**

**Association between selected predictors and the risk of death
180 days after unit-to-unit inter-hospital transfer**

| **S3a. Univariable logistic regression (n=4,327)** |  |  |  |
| --- | --- | --- | --- |
| **Predictor** | **OR** | **95% CI** | **p-value** |
| Days in the ICU before transfer | 1.02 | 1.01 – 1.03 | 0.002* |
| Night-time transfer (reference: day-time) | 0.93 | 0.78 – 1.11 | 0.416 |
| Week-end transfer (reference: weekdays) | 0.98 | 0.85 – 1.13 | 0.755 |
| Distance, per kilometer (km) | 0.99 | 0.99 – 1.00 | 0.019* |
| Long transfer, >25 km (reference: short <25 km) | 0.89 | 0.77 – 1.02 | 0.105 |
| Capacity transfer (reference: clinical) | 1.44 | 1.25 – 1.67 | <0.001* |
| Capacity transfer (reference: repatriation) | 1.28 | 1.06 – 1.54 | 0.010* |
| SAPS3 receiving ICU, per point | 1.08 | 1.07 – 1.09 | <0.001* |
| ICU; Intensive Care Unit, SAPS3; Simplified Acute Physiology Score 3  *denotes a significant p-value (<0.1) | | | |
|  |  |  |  |
| **S3b. Multivariable logistic regression adjusted for ICD-10 diagnosis and SMR**  **in the receiving ICU (n=4,242; 85 missing)** | | | |
| **Variable** | **OR** | **95% CI** | **p-value** |
| Days in the ICU before transfer | 1.01 | 0.99 – 1.02 | 0.328 |
| Distance, per kilometer (km) | 1.00 | 0.99 – 1.00 | 0.765 |
| Capacity transfer (reference: clinical) | 1.12 | 0.92 – 1.36 | 0.266 |
| Capacity transfer (reference: repatriation) | 1.13 | 0.89 – 1.43 | 0.304 |
| SAPS3 receiving ICU, per point | 1.08 | 1.07 – 1.09 | <0.001* |
| SMR; Standardised Mortality Rate, ICU; Intensive Care Unit, SAPS3; Simplified Acute Physiology Score 3, *denotes a significant p-value (<0.05) | | | |

**Table S4a**

**Association between selected predictors and the risk of death**

**90 days after unit-to-unit inter-hospital capacity transfer**

| **Univariable logistic regression (n=1,352)** |  |  |  |
| --- | --- | --- | --- |
| **Predictor** | **OR** | **95% CI** | **p-value** |
| Days in the ICU before transfer | 1.01 | 0.99 – 1.03 | 0.338 |
| Night-time transfer (reference: day-time) | 0.91 | 0.66 – 1.24 | 0.540 |
| Weekend transfer (reference: weekday) | 1.02 | 0.79 – 1.31 | 0.903 |
| Distance, per kilometer (km) | 1.00 | 0.99 – 1.00 | 0.878 |
| Long transfer, >25 km (reference: short <25 km) | 0.93 | 0.74 – 1.17 | 0.535 |
| SAPS3 receiving ICU, per point | 1.06 | 1.05 – 1.07 | <.0.001* |
| ICU; Intensive Care Unit, SAPS3; Simplified Acute Physiology Score 3  *denotes a significant p-value (<0.1) | | | |

**Table S4b**

**Association between selected predictors and the risk of death**

**180 days after unit-to-unit inter-hospital capacity transfer**

| **Univariable logistic regression (n=1,352)** |  |  |  |
| --- | --- | --- | --- |
| **Predictor** | **OR** | **95% CI** | **p-value** |
| Days in the ICU before transfer | 1.01 | 0.99 – 1.03 | 0.268 |
| Night-time transfer (reference: day-time) | 0.91 | 0.67 – 1.24 | 0.536 |
| Weekend transfer (reference: weekday) | 1.07 | 0.83 – 1.37 | 0.603 |
| Distance, per kilometer (km) | 1.00 | 0.99 – 1.00 | 0.963 |
| Long transfer, >25 km (reference: short <25 km) | 0.92 | 0.73 – 1.14 | 0.437 |
| SAPS3 receiving ICU, per point | 1.07 | 1.06 – 1.08 | <.0.001* |
| ICU; Intensive Care Unit, SAPS3; Simplified Acute Physiology Score 3  *denotes a significant p-value (<0.1) | | | |

**Table S5**

**Association between ICD-10 diagnoses and the risk of death
30 days after unit-to-unit inter-hospital transfer**

Multivariable logistic regression with Infection/sepsis as the reference group
n=4,242 (85 missing)

| **ICD-10 diagnosis in the recieving ICU** | **OR** | **95% CI for OR** | **p-value** |
| --- | --- | --- | --- |
| Malignancy | 2.09 | 1.05 - 4.16 | 0.035 |
| Hematology | 2.49 | 1.04 - 5.94 | 0.041 |
| Endocrinal disease | 0.46 | 0.18 - 1.19 | 0.11 |
| Intoxication | 0.21 | 0.08 - 0.53 | <.001 |
| Neurological disorder | 0.72 | 0.46 - 1.13 | 0.151 |
| Cardiac disease | 1.21 | 0.80 - 1.83 | 0.373 |
| Cardiac arrest | 3.69 | 2.74 - 4.97 | <.001 |
| Subarachnoid haemorrhage | 0.72 | 0.45 - 1.16 | 0.174 |
| Cerebrovascular event | 1.67 | 1.22 - 2.28 | 0.001 |
| Aortic rupture/dissection | 1.22 | 0.69 - 2.15 | 0.494 |
| Peripheral arterial disease | 4.24 | 1.86 - 9.69 | <0.001 |
| Shock, undefined | 1.44 | 0.67 - 3.06 | 0.35 |
| Respiratory infection, including pneumonia | 0.84 | 0.62 - 1.15 | 0.284 |
| Airway disorder | 0.44 | 0.17 - 1.13 | 0.088 |
| COPD/asthma/other respiratory disease | 1.53 | 1.16 - 2.02 | 0.003 |
| Acute renal failure/urological disease | 1.26 | 0.74 - 2.14 | 0.401 |
| Acute abdomen | 1.21 | 0.85 - 1.72 | 0.29 |
| Liver failure | 2.65 | 1.60 - 4.38 | <0.001 |
| Pancreatitis/cholecystitis | 0.97 | 0.52 - 1.80 | 0.914 |
| Trauma | 0.38 | 0.27 - 0.55 | <0.001 |
| Surgical complications | 1.00 | 0.59 - 1.70 | 0.991 |
| Isolated traumatic brain injury | 0.86 | 0.61 - 1.21 | 0.392 |
| Postoperative care, n (%) | 0.39 | 0.14 - 1.11 | 0.077 |

**Table S6**

# List of intensive care units (ICU) with geographical coordinates and hospital names

| *Intensive care unit* | | *Latitude* | *Longitude* | *Hospital* |
| --- | --- | --- | --- | --- |
| Alingsås | 57,92881443 | | 12,52113928 | Alingsås hospital |
| Arvika | 59,66530699 | | 12,61577177 | Arvika hospital |
| Bollnäs | 61,35353702 | | 16,36160812 | Bollnäs hospital Aleris |
| Borås | 57,72462172 | | 12,96225417 | Södra Älvsborgs hospital |
| Danderyd | 59,39277088 | | 18,03850198 | Danderyds hospital |
| Eksjö | 57,6662259 | | 14,96509699 | Eksjö hospital |
| Ersta | 59,3168337 | | 18,08914421 | Ersta hospital |
| Eskilstuna | 59,36350168 | | 16,53460016 | Eskilstuna hospital |
| Falun | 60,60966546 | | 15,64337111 | Falun hospital |
| Gällivare | 67,13068108 | | 20,6861067 | Gällivare hospital |
| Gävle | 60,67723935 | | 17,11783323 | Gävle hospital |
| Halmstad | 56,68146168 | | 12,84879721 | Hallands hospital Halmstad |
| Helsingborg | 56,04615108 | | 12,7041809 | Helsingborgs hospital |
| Hudiksvall | 61,72964147 | | 17,09911672 | Hudiksvall hospital |
| Jönköping | 57,76635764 | | 14,19502894 | Ryhov Jönköping hospital |
| K Huddinge IVA | 59,2212671 | | 17,93698181 | Karolinska university hospital Huddinge |
| K Solna BIVA | 59,34845176 | | 18,03065411 | Karolinska university hospital Solna |
| K Solna ECMO | 59,34845176 | | 18,03065411 | Karolinska university hospital Solna |
| K Solna IVA | 59,34845176 | | 18,03065411 | Karolinska university hospital Solna |
| K Solna NIVA | 59,34845176 | | 18,03065411 | Karolinska university hospital Solna |
| K Solna TIVA | 59,34845176 | | 18,03065411 | Karolinska university hospital Solna |
| Kalix | 65,85453412 | | 23,15731488 | Kalix hospital |
| Kalmar | 56,65768339 | | 16,33206025 | Kalmar hospital |
| Karlskoga | 59,31530299 | | 14,50534253 | Karlskoga hospital |
| Karlskrona IVA | 56,18216827 | | 15,60582582 | Blekinge Karlskrona hospital |
| Karlstad | 59,37507917 | | 13,47961977 | Karlstad central hospital |
| Kristianstad | 56,0301558 | | 14,17370241 | Kristianstad central hospital |
| Kungälv | 57,87875981 | | 11,96905765 | Kungälv hospital |
| Lidköping | 58,49755682 | | 13,15205665 | Skaraborgs hospital Lidköping |
| Lindesberg | 59,6021703 | | 15,21700972 | Lindesbergs hospital |
| Linköping BRIVA | 58,40014189 | | 15,62008166 | University hospital Linköping |
| Linköping IVA | 58,40014189 | | 15,62008166 | University hospital Linköping |
| Linköping NIVA | 58,40014189 | | 15,62008166 | University hospital Linköping |
| Linköping TIVA | 58,40014189 | | 15,62008166 | University hospital Linköping |
| Ljungby | 56,83504099 | | 13,93498887 | Ljungby hospital |
| Lycksele | 64,585395 | | 18,67944602 | Lycksele hospital |
| Mora | 61,0163394 | | 14,58440776 | Mora hospital |
| Norrköping | 58,56334353 | | 16,17846964 | Vrinnevi hospital Norrköping |
| Norrtälje | 59,75787588 | | 18,68997451 | Norrtälje hospital |
| NU Trollhättan | 58,31854792 | | 12,2654039 | Norra Älvsborgs hospital |
| Nyköping | 58,75967255 | | 16,99505205 | Nyköping hospital |
| Piteå | 65,31302673 | | 21,49602628 | Piteå älvdals hospital |
| Skellefteå | 64,75635612 | | 20,94283583 | Skellefteå hospital |
| Skövde | 58,42664634 | | 13,85132307 | Skaraborgs hospital Skövde |
| Sollefteå | 63,1745026 | | 17,23481455 | Sollefteå hospital |
| St Göran | 59,33420659 | | 18,0210916 | Capio S:t Görans hospital |
| SU BIVA | 57,68241595 | | 11,96123332 | Sahlgrenska university hospital |
| SU CIVA | 57,68241595 | | 11,96123332 | Sahlgrenska university hospital |
| SU Mölndal | 57,661196 | | 12,01218686 | Mölndal hospital – Sahlgrenska |
| SU NIVA | 57,68241595 | | 11,96123332 | Sahlgrenska university hospital |
| SU TIVA | 57,68241595 | | 11,96123332 | Sahlgrenska university hospital |
| SU Östra | 57,7216578 | | 12,04967345 | Östra hospital - Sahlgrenska |
| SU Östra Inf | 57,7216578 | | 12,04967345 | Östra Sjukhuset - Sahlgrenska |
| Sunderby | 65,67170837 | | 21,93173514 | Sunderby hospital |
| Sundsvall | 62,4086749 | | 17,30596392 | Sundsvall hospital |
| SUS Lund BIVA | 55,71177718 | | 13,19810845 | Skåne university hospital Lund |
| SUS Lund IVA | 55,71177718 | | 13,19810845 | Skåne university hospital Lund |
| SUS Lund NIVA | 55,71177718 | | 13,19810845 | Skåne university hospital Lund |
| SUS Lund TIVA | 55,71177718 | | 13,19810845 | Skåne university hospital Lund |
| SUS Malmö Inf | 55,58973148 | | 13,00070757 | Skåne university hospital Malmö |
| SUS Malmö IVA | 55,58973148 | | 13,00070757 | Skåne university hospital Malmö |
| Södertälje | 59,19869567 | | 17,63210341 | Södertälje hospital |
| SÖS IVA | 59,30954368 | | 18,05594014 | Södersjukhuset hospital Stockholm |
| SÖS MIVA | 59,30954368 | | 18,05594014 | Södersjukhuset hospital Stockholm |
| Torsby | 60,13748847 | | 12,9984676 | Torsby hospital |
| Umeå IVA | 63,81716772 | | 20,29892812 | Norrland university hospital Umeå |
| Umeå TIVA | 63,81716772 | | 20,29892812 | Norrland university hospital Umeå |
| Uppsala BIVA | 59,84858666 | | 17,64022029 | Akademiska Uppsala |
| Uppsala BRIVA | 59,84858666 | | 17,64022029 | Akademiska Uppsala |
| Uppsala CIVA | 59,84858666 | | 17,64022029 | Akademiska Uppsala |
| Uppsala NIVA | 59,84858666 | | 17,64022029 | Akademiska Uppsala |
| Uppsala TIVA | 59,84858666 | | 17,64022029 | Akademiska Uppsala |
| Varberg | 57,09916809 | | 12,27706678 | Hallands hospital Varberg |
| Visby | 57,64833049 | | 18,30047709 | Visby hospital |
| Värnamo | 57,17444656 | | 14,02846162 | Värnamo hospital |
| Västervik | 57,75549072 | | 16,63601013 | Västervik hospital |
| Västerås | 59,61740203 | | 16,58159681 | Västmanlands hospital Västerås |
| Växjö | 56,87434886 | | 14,80735702 | Växjö central hospital |
| Ystad | 55,43334311 | | 13,81686271 | Ystad hospital |
| Örebro IVA | 59,27585005 | | 15,22512029 | Örebro university hospital |
| Örebro TIVA | 59,27585005 | | 15,22512029 | Örebro university hospital |
| Örnsköldsvik | 63,29970823 | | 18,71218477 | Örnsköldsviks hospital |
| Östersund | 63,18256764 | | 14,63603081 | Östersund hospital |
